# COMPULS: design of a multicenter phenotypic, cognitive, genetic, and magnetic resonance imaging study in children with compulsive syndromes

**DOI:** 10.1186/s12888-016-1072-6

**Published:** 2016-10-26

**Authors:** Jilly Naaijen, Saskia de Ruiter, Marcel P. Zwiers, Jeffrey C. Glennon, Sarah Durston, David J. Lythgoe, Steven C. R. Williams, Tobias Banaschewski, Daniel Brandeis, Barbara Franke, Jan K. Buitelaar

**Affiliations:** 1 Department of Cognitive Neuroscience, Donders Institute of Brain, Cognition and Behaviour, Radboud University Medical Center, Geert Grooteplein Noord 10 (Huispost 126), 6525 EZ Nijmegen, The Netherlands; 2 Karakter child and adolescent psychiatry university center, Nijmegen, The Netherlands; 3 NICHE lab, department of psychiatry, Brain Center Rudolf Magnus, University Medical Center Utrecht, Utrecht, The Netherlands; 4 Department of Neuroimaging, King’s College London, Institute of Psychiatry, Psychology and Neuroscience, London, UK; 5 Department of Child and Adolescent Psychiatry and Psychotherapy, Central Institute of Mental Health, Medical Faculty, Mannheim/Heidelberg University, Mannheim, Germany; 6 Department of Child and Adolescent Psychiatry, University of Zurich, Zurich, Switzerland; 7 Center for Integrative Human Physiology, University of Zurich, Zurich, Switzerland; 8 Neuroscience Center Zurich, University of Zurich and ETH Zurich, Zurich, Switzerland; 9 Departments of Human Genetics and Psychiatry, Donders Institute of Brain, Cognition and Behaviour, Radboud University Medical Center, Nijmegen, The Netherlands

**Keywords:** Compulsivity, Fronto-striatal circuit, Glutamate, ADHD, ASD, OCD

## Abstract

**Background:**

Compulsivity, the closely linked trait impulsivity and addictive behaviour are associated with several neurodevelopmental disorders, including attention-deficit/hyperactivity disorder (ADHD), autism spectrum disorder (ASD), and obsessive compulsive disorder (OCD). All three disorders show impaired fronto-striatal functioning, which may be related to altered glutamatergic signalling. Genetic factors are also thought to play an important role in the aetiology of compulsivity-related disorders.

**Methods:**

The COMPULS study is a multi-center study designed to investigate the relationship between the traits compulsivity, impulsivity, and, to a lesser extent, addictive behaviour within and across the neurodevelopmental disorders ADHD, ASD, and OCD. This will be done at the phenotypic, cognitive, neural, and genetic level. In total, 240 participants will take part in COMPULS across four different sites in Europe. Data collection will include diagnostic interviews, behavioural questionnaires, cognitive measures, structural, functional and spectral neuroimaging, and genome-wide genetic information.

**Discussion:**

The COMPULS study will offer the unique opportunity to investigate several key aspects of compulsivity across a large cohort of ADHD, ASD and OCD patients.

## Background

Compulsivity and impulsivity are cross-disorder traits that are present across various neurodevelopmental disorders, such as attention-deficit/hyperactivity disorder (ADHD) autism spectrum disorder (ASD) and obsessive compulsive disorder (OCD).

Compulsivity can be defined as the repetitive, irresistible urge to perform certain behaviour, the experience of loss of voluntary control over this intense urge, the diminished ability to delay or inhibit thoughts and behaviours, and the tendency to perform repetitive acts in a habitual or stereotyped manner [1, 2]. Compulsivity is a cross-disorder trait observed in the phenotypically distinct neurodevelopmental disorders ASD and OCD. ASDs have a prevalence of 1.5 % [3, 4] and are characterized by deficits in reciprocal social interaction and communication and by restricted, repetitive and stereotyped patterns of behaviour, interests and activities [5]. OCD, on the other hand, is a relatively common anxiety disorder, characterized by repetitive thoughts, impulses, or images (obsessions), and repetitive behaviours or mental acts (compulsions) that cause marked distress [5]. OCD has its onset in late childhood and is present in about 2.5 % of the adult population [6]. Repetitive behaviours are among the core features of both ASD and OCD, and comparison of symptom characteristics has demonstrated more similarities than differences [7]. In addition to symptom overlap, similar executive function impairments related to inhibiting compulsive behaviours have been reported in first degree relatives of people with ASD and OCD [8]. Compulsivity can be seen as an overarching concept that includes both the failure to resist an impulse, which links it to impulsivity, and maladaptive habitual patterns of behaviours, which relates to addictive behaviour.

Impulsivity is described as a predisposition toward rapid unplanned reactions to internal or external stimuli with diminished regard to the potentially negative consequences of these reactions [2]. Impulsivity is one of the core characteristics of ADHD. ADHD is characterized by clinically significant levels of hyperactivity, impulsivity and/or inattention and is affecting about 5 % of all school-age children world-wide [9, 10]. About 40 % of children with ADHD have comorbid ASD (e.g. [11–13]). Further, prevalence of OCD in children with ADHD is estimated at 8 %, which is a 2-3 fold increase compared to non-ADHD children [7, 14]. There are strong similarities between the uncontrollable behaviour based on impulsivity (seen in ADHD) and the excessive and unwanted rituals related to compulsivity [15].

**Fig. 1:**
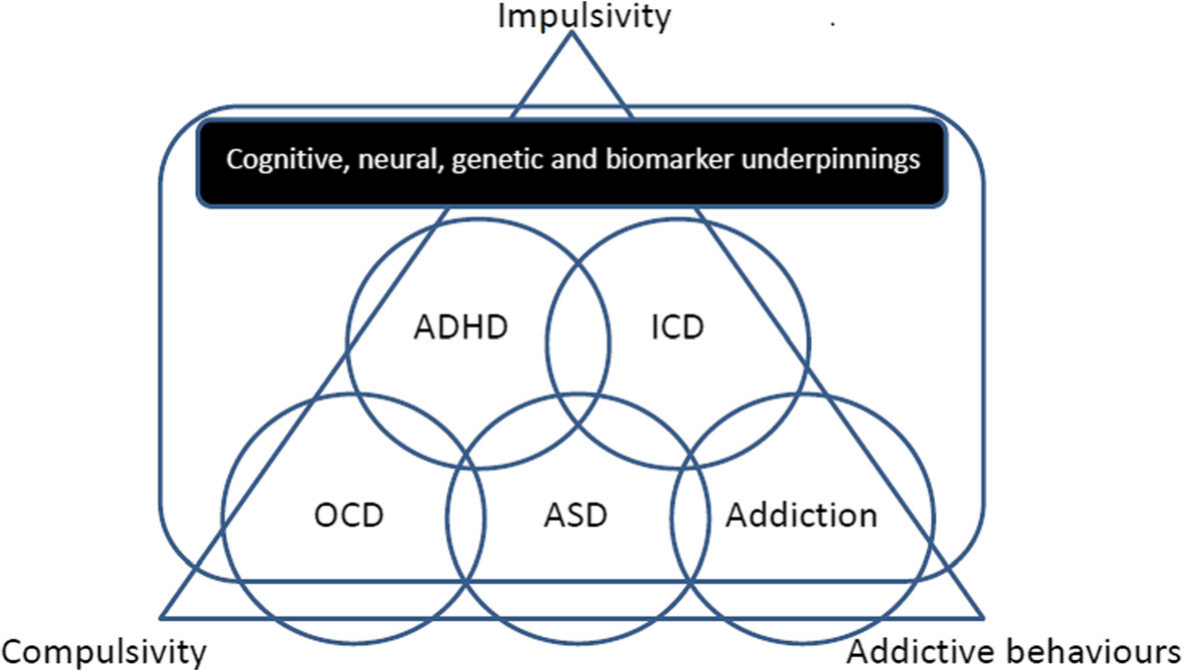
Framework for understanding the relationships between cross-disorder traits impulsivity, compulsivity and addictive behaviours, between discrete disorders, and between traits and disorders by adding a cognitive, neural, genetic and biomarker level of understanding. (ICD, impulse control disorder)

Addictive behaviour is characterized by both impulsivity and compulsivity [16]. One shows compulsive drug-seeking, loss of control in limiting this intake, and the emergence of a negative emotional state reflecting a motivational withdrawal syndrome, when access to the drug is prevented. Impulsivity often dominates early stages of addiction, while compulsivity becomes important in later stages. See Fig. 1 for the relation between compulsivity, impulsivity and addictive behaviour in different disorders.

Separate but intercommunicating cortico-striatal circuits seem to be involved in impulsivity and compulsivity [2, 17]. In the impulsive circuit, a striatal component (nucleus accumbens) may drive impulsive behaviours, and a prefrontal component (anterior cingulate (ACC), ventromedial prefrontal cortex) exerts inhibitory control. Similarly, compulsive behaviour may be driven by a striatal component (caudate, putamen), which is controlled by the orbitofrontal cortex (OFC). Increased activity in the striatum and/or decreased activity in the prefrontal cortex (PFC) may alter the functioning of these cortico-striatal circuits and cause impulsive or compulsive disorders, both characterized by deficits in response inhibition. Although, in contrast to impulsivity, compulsivity is considered a maladaptive perseveration of behaviour that does not fall within the range of normal behaviour [15], both patterns of behaviour often co-occur [18, 19].

Compulsivity-related disorders differ in their age of onset. ASD starts very early in life, ADHD in early childhood, and OCD has its onset in late childhood or early adolescence. This variation in onset of impulsivity/compulsivity may be related to variation in the maturation of the fronto-striatal circuits and the role of glutamate within these circuits. Recent theories suggest that striatal brain regions underlying impulsive and compulsive behaviours may show a nonlinear developmental pattern with a peak inflection between 13 and 17 years of age [20]. The prefrontal regions on the other hand, which are important for top-down regulation of (striatum-driven) impulsive and compulsive behaviour, show a more linear pattern of maturation well into young adulthood.

Glutamate is the major excitatory neurotransmitter in the human brain and is critical to the understanding of the top-down control of the prefrontal cortex over the dorsal and ventral striatum [21]. The impulsivity- and compulsivity-related fronto-striatal circuits are notable for their relatively rich glutamatergic receptor density. Glutamate modulates the neural activity and metabolism of these circuits, as is reflected by the effects of glutamate on synapse induction and elimination as well as synaptic transmission via ionotropic and metabotropic glutamate receptors [22]. Indeed, glutamatergic projections from the prefrontal sub-regions to the striatum are already known to play a key role in various compulsive and impulsive behaviours including repetitive behaviours such as stereotypy seen in ASD, impulsivity seen in ADHD, and feelings of loss of control seen in OCD (for a review, see [23]).

**Fig. 2:**
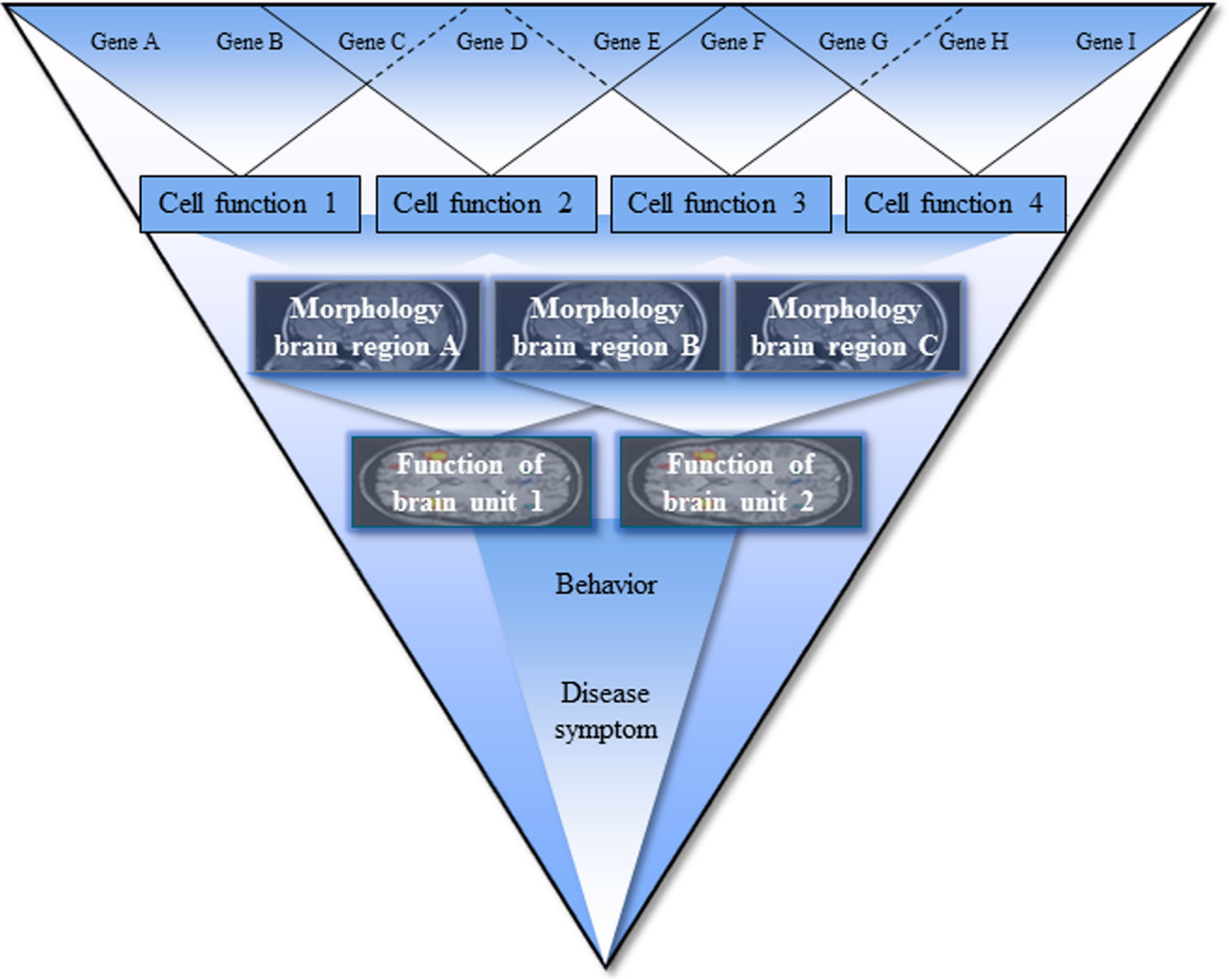
Simplified representation of relation between genes, cell functioning, brain functioning and behaviour. Many genes are involved in causing disease symptoms, but reduced numbers of genes are involved in features associated with the disease symptoms, like brain functioning and cell functioning (Adapted from [64])

The study of genetics offers an opportunity to gather additional evidence for the role of glutamate. Many compulsivity- and impulsivity-related disorders are substantially heritable but genetically very complex, with multiple genetic factors of varying penetrance implicated [10, 24, 25]. A number of candidate genes have been identified, suggesting glutamatergic pathways to operate in ASD, ADHD, and OCD. For example, variation in genes encoding the glutamate transporters *SLC1A1, SLC1A2,* and *SLC1A3* are strong candidate genes for both ASD and OCD [26]. In addition, genes encoding the NMDA receptors *GRIN2A* and *GRIN2B* have been implicated in ASD ([27], and *GRIN2B* has also been associated with ADHD [28] and OCD [29]. While glutamate has an important role in the neurobiology of compulsivity and impulsivity and the related disorders, it is certainly not the only biological substrate involved. For example, we have recently identified central insulin signalling as an additional molecular cascade involved in OCD [30] and neurite outgrowth as a neurodevelopmental process implicated in ADHD [31] and ASD [32]. Genetic variation in such genes is likely to alter their regulation and/or function, leading to changes in the encoded proteins and biological processes contributing to proper cell function. Brain structure and brain function, also highly heritable, may mediate the effects of the variation in genes and proteins on compulsive and impulsive behaviours and related disorders [32, 33]. See Fig. 2 for a representation of this relation between genes, cell functioning, brain and behaviour.

## Methods/Design

### Aims of the study

COMPULS is a multicenter study as part of the overarching TACTICS study (http://www.tactics-project.eu) investigating the relationship between the traits compulsivity, impulsivity, and - to a lesser extent - addictive behaviour within and across the neurodevelopmental disorders ASD, ADHD, and OCD. This will be the first study to integrate these different traits and disorders into one design. The primary objectives are to examine whether these traits are related to structural and functional connectivity of the fronto-striatal circuits and whether these behavioural traits are predicted by or related to abnormal glutamatergic concentrations in these fronto-striatal circuits. Secondary objectives of COMPULS are to explore the role of candidate genes or candidate genetic pathways involved in compulsivity, and related traits within these disorders.

These objectives will be investigated at the phenotypic, cognitive, neural, and genetic level in a prospective longitudinal design. This paper describes the design, measures, and rationale of COMPULS.

### Participants

Data collection occurs at four different sites across Europe (Radboud university medical center and the Donders Institute for Brain, Cognition and Behaviour, Nijmegen, The Netherlands; Brain Centre Rudolf Magnus, University Medical Centre Utrecht, Utrecht, The Netherlands; King’s College London, London, United Kingdom, and Central Institute of Mental Health, Medical Faculty Mannheim, University of Heidelberg Mannheim, Germany). We include 60 participants with ASD, 60 with OCD, 60 with ADHD, and 60 healthy comparison participants in the age range of 8 to 12 years. Every site includes 15 ASD, OCD, and healthy comparison participants (adding up to a total of 60 per group). The ADHD sample is completely collected in Nijmegen. The total sample size consists of 240 participants. Setting alpha at .05 (two-tailed) our sample size of *N* = 240 has 80 % statistical power to detect a beta (standardized regression coefficient) of 0.21. This sample size allows for about 15 covariates/predictors in total. Thus our sample size will provide us sufficient power to establish predictors of small effect size. A group size of 60 participants per group (OCD, ASD, ADHD, controls) further allows us to examine the disease modifying effects in planned contrasts in these regression models (dummy variables).

Inclusion criteria for all groups are age between 8 and 12 years old, IQ > 70, ability to speak and comprehend the native language of the country in which the assessment takes place, and a signed informed consent by parents or legal representatives. For the diagnosis groups, a DSM-IV-TR or DSM-5 diagnosis of the respective disorder has to be present. Exclusion criteria for the ASD, OCD, and ADHD children are diagnoses of the other disorders (comorbidity), e.g. an OCD diagnosis in an ASD participant. Other exclusion criteria include IQ < 70, major physical illness of the vascular, endocrine, pulmonic or the gastrointestinal system, all contra-indications for MR assessment, such as the presence of metal objects in the body (i.e. pacemaker, dental braces), and a history or presence of neurological disorders. For the healthy comparison participants, no first degree family members are allowed to have any psychiatric disorder.

### Measures

#### Diagnosis

To determine the diagnoses several interviews will be administered, depending on the symptoms of the participant. The autism diagnostic interview-revised (ADI-R [34]) is a structured developmental interview administered to the parent(s) to assess the symptoms of ASD and make an ASD DSM-IV-TR diagnosis in the child. For an ADHD diagnosis, the semi-structured Kiddie Schedule for Affective Disorders and Schizophrenia (K-SADS [35]) will be administered to the parent(s). The Children’s Yale Brown Obsessive Compulsive Scale (CYBOCS [36]) is used to interview the parent(s) and child for the presence of obsessions and/or compulsions and symptom severity, when OCD is present. This interview will be performed in all participant groups when screening questions confirm the presence of obsessions or compulsions. In addition, all parents are interviewed using the structured Diagnostic Interview Schedule for Children (DISC [37]), the Development and Well-being Assessment (DAWBA [38]) or the K-SADS to assess the presence of possible comorbidities such as oppositional defiant disorder, conduct disorder, and the presence of tics/Tourette’s syndrome and anxiety disorders. The diagnostic tools provide operational definitions of individual symptoms as well as diagnosis-relevant questions, such as onset of symptoms and impairment in several areas of life.

#### Questionnaires

Questionnaires are used to assess (a) symptom severity of possible comorbid disorders, such as ADHD (Conners’ Parent Rating Scale (CPRS R:L [39]) and ASDs (Children’s Social Behavioural Questionnaire (CSBQ [40]), (b) Substance use disorders (SUDs; Alcohol Use Disorders Identification Test (AUDIT [41]), drug abuse (Drug Abuse Screening Test (DAST [42]), and nicotine dependence (Fagerstrøm Test for Nicotine Dependence (FTND [43]), (c) lifetime alcohol-related problems (Michigan Alcohol Screening Test (MAST [44]), (d) gambling problems (South Oaks Gambling Screen (SOGS [45]), (e) repetitive behaviour (Repetitive Behaviour Scale-Revised (RBS-R [46]), (f) emotional and behavioural problems (Child Behaviour Check List (CBCL [47]); Teacher report form (TRF) [47]), and (g) physical development [48], to determine the developmental stage at the time of assessment. All questionnaires are completed by the parent(s). A final set of measures is taken to determine patterns of use of prescribed medication (in house self-report for medication use).

#### Cognitive assessment

All children complete an extensive protocol of cognitive tasks measuring (a) intellectual functioning estimated from the (i) vocabulary, (ii) block design, (iii) similarities, and (iv) picture completion subtests of the WISC [49], (b) motor inhibition, (c) cognitive flexibility, and (d) motor speed. Except for the subtests of the WISC, all tests are computerized. For cognitive flexibility, a timing task will be administered [48, 49]. For measuring motor inhibition and motor speed, we will use the set-shifting and baseline-speed tasks of the Amsterdam Neuropsychological Tests (ANT [50]).

#### MR measurements

Participating children complete a brain scan session in a magnetic resonance imaging (MRI) scanner. At the four different sites, comparable 3 Tesla MRI scanners are used (Siemens Trio and Siemens Prisma, Siemens, Erlangen, Germany; Philips 3 T Achieva, Philips Medical Systems, Best, The Netherlands; General Electric MR750, GE Medical Systems, Milwaukee, WI, USA) using a 32 channel (Siemens) or 8 channel head coil (Philips, GE). A scanning session includes an anatomical T1 scan, resting state functional MRI (R-fMRI), Diffusion Tensor Imaging (DTI), and two functional imaging tasks including a behavioural inhibition (Stop) task [51] and a shortened monetary reward anticipation task [50, 51]. COMPULS’ areas of interest are the fronto-striatal circuits, important signalling regions that are undergoing developmental changes in the age range of 8 to 12 years. As described in the introduction, differences exist in the onset of compulsive disorders. In order to capture glutamatergic deficits across these disorders, an MR spectroscopy session with two voxels of interest encompassing a part of these fronto-striatal circuits (left dorsal striatum and ACC) is also included in the MRI session.

The structural T1 scanning sequences are based on the ADNI GO protocols [52–55] to be matched as closely as possible across the different scanning sites. For R-fMRI we use a multi-echo sequence to be able to separate BOLD from non-BOLD signal more accurately [56], DTI acquisition is chosen to better resolve crossing fibers and task-based fMRI acquisition is designed for optimal signal stability and homogeneity over the different sites (see Table 1 for an overview of the scan sequences). In order to perform quality assessment of the MR scanning sessions across four different sites, we will make use of phantoms and so-called ‘travelling heads’. By using phantoms, we can assess subtle changes in scanner output over time (due to scanner drifts, system upgrades, etc.), but we feel that this is not sufficient to allow for calibration of human brain data. In order to compare the quality of the human brain scans across sites, the travelling heads are thus used, i.e. three adult volunteers will travel to the four participating sites at the start of COMPULS, at the end of the data collection, and in-between, whenever scanner upgrades are performed. This will allow us to assess inter-scanner reliability and take into account differences between scanners. These travelling head data-sets will additionally be used to compare intra- and inter-site variability with regard to the measurement of glutamate. On-site MRS training will be provided before data-collection to assure similarity in terms of voxel placement across sites.

**Table 1:** Scan sequences

Sequence	Site	TR/TE/TI (ms)	Flip angle	Field of view (mm)	Matrix RL/AP/slices	Voxel-size (mm)	Gap (%)	Parallel Imaging	*b* value	Directions/*b*0’s	Averages Water suppressed/unsuppressed
T1	Nijmegen *(Siemens)*	2300^*^/2.98/900	9	256	212/256/176	1.0 * 1.0 * 1.2	NA	2	NA	NA	NA
	Mannheim *(Siemens)*	2300^*^/2.98/900	9	270	212/254/176	1.1 * 1.1 * 1.2	NA	2	NA	NA	NA
	Utrecht *(Philips)*	6.8^*^/3.10/823	9	270	204/252/170	1.1 * 1.1 * 1.2	NA	1.8	NA	NA	NA
	London *(GE)*	7.31^*^/3.02/400	11	270	256/256/196	1.1 * 1.1 * 1.2	NA	1.75	NA	NA	NA
MRS PRESS	All	3000/30/-	NA	NA	NA	20 * 20* 20	NA	NA	NA	NA	96/16
R-FMRI^#^	All	2300/12^a^–13^b^/-	80	240	240/240/33	3.8 * 3.8 * 3.8	11	2 – 2.5^c^	NA	NA	NA
Functional tasks	All	2070/35/-	74	192	192/192/36	3.0 * 3.0 * 3.0	13	2	NA	NA	NA
DTI	All	12000/103/-	90	256	256/256/72	2.0 * 2.0 * 2.0	0	2	1500	60/2	NA

^*^As provided by the manufacturer. Philips and GE define a TR as the time an excitation pulse is given, while Siemens defines TR as the time between inversion recovery pulses a volume

^#^Multi-echo resting state fMRI: TE2 is 31 ms for London and Utrecht, 29 for Mannheim and 28.41 for Nijmegen. TE3 is 48 for London, 49 for Utrecht, 46 for Mannheim and 44.82 for Nijmegen

^a^Nijmegen, Mannheim

^b^Utrecht, London

^c^Utrecht

#### Genetic determinants

Participants will provide 40 ml blood for DNA-isolation and assessment of biomarkers for which serum and plasma fractions will be prepared. The venapuncture will be performed by a trained practical nurse. If a participant refuses venapuncture, saliva will be used for DNA-isolation instead.

DNA, RNA, and plasma/serum fractions will be isolated from blood/saliva using standard techniques and stored at the department of Human Genetics of the Radboud university medical center. MicroRNAs (miRNA) will be extracted from total RNA isolated from blood collected in PAX-gene tubes. The DNA will be subjected to genome-wide genotyping, providing a basis for the computation of polygenic risk scores; such scores will be incorporated in analyses of disease risk, and might improve phenotypic prediction [57]. The current study on itself will have a small sample size and is of course insufficient to detect genome-wide significant effects. However, by aggregation of multiple variants at the level of individual genes or gene-sets through mass-univariate or multivariate methods, power may be increased [58–60]. This will provide opportunities for candidate gene/pathway analyses, which of course will require appropriate correction for multiple testing. Lastly, by contributing to international collaborations like the Psychiatric Genomics Consortium (PGC; [61] and Enhancing Neuro Imaging Genetics through meta-analysis (ENIGMA; [62])), our data may contribute to genome-wide gene-finding studies will become more powerful.

Protein and miRNA levels of blood-expressed candidate genes will be monitored. In the case of proteomics, this will involve using multiplex immunoassay profiling of serum. In addition, determination of glutamate, serotonin, and insulin levels will be performed. This work will be performed in the laboratory of Professor Bahn in Cambridge.

#### Somatic and other measures

To obtain an estimate of possible abnormal growth or other physiological abnormalities, we measure body length, weight, waist circumference, and ask for the presence of allergies or food restrictions.

### Procedures

#### Assessment

After initial contact through information packages (including general project information) sent by post, the parents are phoned to check interest in participation. In case of interest, a brief screening will be conducted to control for possible exclusion criteria. If the child meets the inclusion criteria, a questionnaire package will be sent via regular post, also including informed consent/assent forms and general information about the test-location.

If feasible according to the child’s capability, one testing day is organized covering all assessments. During this day, parents will be assessed with the interview. If screening questions are answered positively, screening will be followed by the full supplementary module of the specific disorder. When applicable, CYBOCS and/or the ADI is administered. Cognitive tests with the child will be administered in a fixed order and - due to the length of the battery - split in two parts, part A and part B. The order of administration of the two parts will be counterbalanced across children. Before participating in the MR session, children will be prepared for scanning using a dummy scanner. In this dummy session, children are presented with MR sounds, the button box needed for task completion, and lying in a tight environment. In addition, time to practice the MR tasks is provided during this dummy session. Should a child (or his/her parent) report anxiety to enter the MR scanner, the session will be ended. The anxiety is monitored by using a visual analogue scale (VAS), a scale from 1 to 10, on which the child, parent, and researcher rate the anxiety (1 means no anxiety and 10 means very high anxiety). If the score is 8 or higher, the scan will not be performed. After the MR assessment, the blood-sample will be taken by trained professionals for biological analyses.

A monetary reward is granted and travel costs will be reimbursed. Children who complete the MR session (or at least the anatomical session) are provided with a picture of their anatomical MR scan. Moreover, all children receive an extra monetary reward, which they can gain during the cognitive assessments (inside or outside the MR scanner), and a short report of their performance on the IQ tests, if requested.

#### Follow up

A second wave data collection procedure will be performed after the first with an interval of at least 1 year. The same measurements will be administered, except for the ADI-R interview. These two time-points together can give insights in development during an important phase in life, when the transition from childhood and adolescence takes place. The fronto-striatal circuit undergoes several changes during development [63], which we can map longitudinally with this study-design.

#### Staff training and supervision

The cognitive testing, diagnostic interview, and MRI scanning are restricted to trained personnel. This includes training in Good Clinical Practice (GCP). For the diagnostic interview, staff members have to attend interview sessions led by a trained interviewer. When practicing interviews, one will be under supervision of a clinician or trained professional. The ADI-R is an interview that requires an official certificate and will only be administered by those officially trained. For the diagnostic interviews quality control meetings will be held to discuss controversial cases and to maintain agreement.

To standardize cognitive testing and neuroimaging sessions as much as possible, written standard operating procedures (SOPs) were developed for administration of cognitive tests and MR assessments. All researchers are trained to administer the test battery using the SOPs and under supervision before they can administer tests on their own. The MRI training consists of practicing to operate the scanner computer, learning security procedures, and monitoring quality of the data (i.e. motion artefacts, spike identification).

#### Data management and quality control

Every participant is coded with an anonymous identifier number to separate personal data from scientific data. Data acquisition will be documented in a case report listing all data available for that person. Notes regarding factors that may influence the data (analysis) are provided. Every note, questionnaire, informed consent form, etc. will be kept together in a dossier. All digital data will be stored on the device, on which it is administered (laptops), and then securely uploaded to a central storage server. This server backs-up to tape daily. Researchers at the Radboud university medical center are also obligated to archive raw data on at least two different archiving disks.

All data, except for the MRI data, will be uploaded to a central SQL (Structured Query Language) database. This database meets the acquired safety regulations per participating site, and only assigned researchers will have access across the four sites. Data integrity will be controlled by comparing uploaded data to descriptions in the dossier. For MRI data, quality checks are also performed. T1 anatomical scans are quality-rated on a 4-point scale, MR spectra undergo visual inspection for each participant, and various quantitative parameters are calculated for the functional and diffusion imaging data, such as spatial and temporal signal-to-noise-ratios and realignment parameters.

## Discussion

The COMPULS database will offer the unique opportunity to study several key aspects of compulsivity in a large cohort of ASD, ADHD, and OCD patients. By assessing neural and cognitive systems during the critical period of transition from childhood to adolescence across disorders, we can investigate stability and changes of neural systems, which seem to be critical in the development of compulsive behaviour. Integrating data from cognitive, neural, and genetic markers linked to compulsivity can largely increase our understanding of neural mechanisms involved in compulsivity and related disorders. Additionally, by using a dimensional approach of compulsive and impulsive behaviour in several different disorder groups, we can find differing and overlapping deficits, which may explain the high comorbidity rates. At the clinical level, this would provide means for identifying children at risk for poor clinical outcome and provide a basis for the development of better treatment strategies that take into account this dimensional nature of neurodevelopmental disorders. COMPULS will contribute its data to meta- and mega-analyses in international initiatives like the Psychiatric Genomics Consortium [61] and the ENIGMA Consortium [62] and will collaborate with other large international project with a focus on neurodevelopmental disorders (i.e. EU AIMS: http://www.eu-aims.eu/).

At the more technical and logistic level, the COMPULS database will provide opportunities to thoroughly investigate the effect of acquiring data at different centers on outcome variables during analyses. It can examine which types of data are most sensitive to the effect of multi-centre collection, and can thus provide input for future multi-center studies. It will form an international scientific resource, which may be accessed by other researchers in the field on request.

## Abbreviations

(f)MRI: (functional) magnetic resonance imaging
ACC: Anterior cingulate cortex
ADHD: Attention deficit hyperactivity disorder
ASD: Autism spectrum disorder
DSM: Diagnostic and statistical manual of mental disorders
DTI: Diffusion tensor imaging
MRS: Magnetic resonance spectroscopy
OFC: Orbito-frontal cortex
PFC: Prefrontal cortex
